# Critical view of safety in laparoscopic cholecystectomy: A prospective investigation from both cognitive and executive aspects

**DOI:** 10.3389/fsurg.2022.946917

**Published:** 2022-08-01

**Authors:** Yi Jin, Runwen Liu, Yonghua Chen, Jie Liu, Ying Zhao, Ailin Wei, Yichuan Li, Hai Li, Jun Xu, Xin Wang, Ang Li

**Affiliations:** ^1^ Department of Pancreatic Surgery, West China Hospital, Sichuan University, Chengdu, China; ^2^ Department of Algorithm, Chengdu Withai Innovations Technology Company, Chengdu, China; ^3^ National Chengdu Center for Safety Evaluation of Drugs, West China Hospital, Sichuan University, Chengdu, China; ^4^ Department of Science and Technology, Guang'an People's Hospital, Guang'an, China; ^5^ Department of Hepatobiliary Surgery, Guang'an People's Hospital, Guang'an, China; ^6^ Department of Hepatobiliary Surgery, Chongzhou People's Hospital, Chengdu, China; ^7^ Department of Minimal Invasive Surgery, Shangjin Nanfu Hospital, Chengdu, China

**Keywords:** laparoscopic cholecystectomy (LC), critical view of safety (CVS), cognition, execution, surgical safety

## Abstract

**Background:**

The achievement rate of the critical view of safety during laparoscopic cholecystectomy is much lower than expected. This original study aims to investigate and analyze factors associated with a low critical view of safety achievement.

**Materials and Methods:**

We prospectively collected laparoscopic cholecystectomy videos performed from September 2, 2021, to September 19, 2021, in Sichuan Province, China. The artificial intelligence system, SurgSmart, analyzed videos under the necessary corrections undergone by expert surgeons. Also, we distributed questionnaires to surgeons and analyzed them along with surgical videos simultaneously.

**Results:**

We collected 169 laparoscopic cholecystectomy surgical videos undergone by 124 surgeons, among which 105 participants gave valid answers to the questionnaire. Excluding those who conducted the bail-out process directly, the overall critical view of safety achievement rates for non-inflammatory and inflammatory groups were 18.18% (18/99) and 9.84% (6/61), respectively. Although 80.95% (85/105) of the surgeons understood the basic concept of the critical view of safety, only 4.76% (5/105) of the respondents commanded all three criteria in an error-free way. Multivariate logistic regression results showed that an unconventional surgical workflow (OR:12.372, *P* < 0.001), a misunderstanding of the 2nd (OR: 8.917, *P* < 0.05) and 3rd (OR:8.206, *P* < 0.05) criterion of the critical view of safety, and the don't mistake “fundus-first technique” as one criterion of the critical view of safety (OR:0.123, *P* < 0.01) were associated with lower and higher achievements of the critical view of safety, respectively.

**Conclusions:**

The execution and cognition of the critical view of safety are deficient, especially the latter one. Thus, increasing the critical view of safety surgical awareness may effectively improve its achievement rate.

## Introduction

Minimally invasive surgery is widely implemented in the surgical field owing to its advantages such as smaller incision, less postoperative pain, and shorter recovery time compared with the laparotomy method (1–4). Laparoscopic cholecystectomy (LC) is currently the gold standard for the treatment of symptomatic gallstone (5). The number of surgical patients who underwent LC in the United States is approximately 750,000–1,000,000 yearly (6). Although LC is a simple and routine surgical procedure, previous studies reported that LC is associated with major bile duct injury (BDI) rates of 0.15%–0.36% and an overall biliary complication rate of 1.5% (7–12). BDI significantly affects the quality of life, life expectancy, and financial situation of patients (13–16). Surgeons bear extreme psychological pressure and face the risk of lawsuit from patients (17, 18). Safe cholecystectomy techniques such as critical view of safety (CVS), intraoperative cholangiography, and bail-out have been proposed to decrease the incidence of BDI (6). The Society of American Gastrointestinal and Endoscopic Surgeons (SAGES) consensus and Tokyo Guidelines 2018 indicated that the achievement of CVS is an effective technique for decreasing BDI (19, 20). The findings from retrospective multicenter studies indicated that a routine achievement of CVS could significantly decrease the incidence of BDI to 2 patients in every 1,000,000 people (21–24).

Previous studies reported that CVS was highly feasible (85%–95%) (21–23). However, the actual achievement rate of CVS was significantly lower than expected (25, 26). A survey of 343 samples obtained from American surgeons showed an initial CVS achievement rate of 15.9% (25). Another study conducted in Stanford University, comprising 1,051 videos obtained from 31 surgeons, showed a CVS achievement rate below 10% (26). In order to increase the achievement rate of CVS and decrease the incidence of BDI, Nakazato et al. (27) requested every participating surgeon to perform four LC procedures recorded: twice before and twice after a curriculum focused on CVS, which indicated that a structured curriculum on achieving a quality CVS improved their frequency of achieving CVS during LC. In addition, Mascagni et al. (25) performed a short intraoperative time-out, and the results showed that time-out was associated with an improved CVS achievement rate. Although the application of the above methods had significantly improved the achievement rate of CVS, there were still more than half of LC surgeries without routine CVS. Therefore, it is imperative to comprehensively explore the factors that affect the further improvement of CVS. Nevertheless, due to the complex nature of causes affecting CVS achievement, including both cognitive and executive factors, there is still a lack of this type of study.

Therefore, the current study sought to achieve the following two aims through reviewing and analyzing results obtained from a recently organized multi-institution artificial intelligence–assisted LC video competition: (1) to evaluate the cognition of surgeons in terms of surgical safety from questionnaire and explore the executive status from corresponding surgical videos; (2) to explore factors affecting the achievement of CVS from both cognitive and executive aspects.

## Materials and methods

### Collection and analysis of surgical videos

A multi-institution LC surgical video competition was launched in the southwest regions of China (Sichuan Province) at the beginning of September 2021. The recruitment criteria for participation were as follows: (1) videos collected prospectively between September 2, 2021, and September 19, 2021; (2) The videos should comprise one and only complete LC procedure; (3) The surgical video used in the competition must be performed by the participant; (4) The videos were further assigned to an inflammatory group or a non-inflammatory group according to the Parkland Grading System (28).

SurgSmart, an intelligent surgical quality control system that could automatically recognize surgical phases, inflammatory status, critical division actions and CVS status, was used to analyze all the participating videos. Three well-trained experts reviewed the results and made necessary corrections on the customized platform (https://www.withai.com/events/202109-completion/) to ensure the accuracy of the analysis results by SurgSmart. Videos with a Parkland score ≥3 were assigned to the inflammatory group, whereas videos with a Parkland score <3 were assigned to the non-inflammatory group. The LC procedure was divided into seven surgical phases—establish access (EA), adhesion lysis (AL), mobilize hepatocystic triangle (MHT), dissect gallbladder from liver bed (DGB), extract the gallbladder (EG), clear the operative region (COR), and idle time (ID)—based on our previous study (29). The ID was defined as the period when the camera was aimed away from the abdomen or indicated no action in the abdomen. CVS was defined by Strasberg et al. (30) for the first time and comprised three criteria, namely, C1 (clearly dissecting the hepatocystic triangle before cutting the cystic duct/artery), C2 (dissecting the lower 1/3 of the cystic plate before cutting the cystic duct/artery), and C3 (before cutting the cystic duct/artery, both structures should be clearly dissected) (31). The score system of CVS is presented in Supplementary Table 1. The CVS status was divided into three classes according to the sum score, namely, achieved (5/6), medium (3/4), and low (0/1/2). Supplementary Figure 1 is an example of the surgical report processed by the SurgSmart algorithm, including Parkland score, the identification of surgical phases, and the evaluation of CVS achievement.

The study was conducted in accordance with the Declaration of Helsinki and was approved by the Ethics Committee of West China Hospital of Sichuan University (No. 2022-316).

### Questionnaire survey

All participants received a Web-based survey (https://www.wjx.cn/) on September 20, 2021. Each participant had only one chance to respond to the survey, and surgeons who had not responded before September 21, 2021, were excluded. The survey comprised seven modules as presented in the Appendix in the Supplementary file. Module A investigated participants’ cognition on the appropriate time to complete an LC procedure in both easy and difficult conditions. Module B explored the balance between delicacy and efficiency in each of the surgical phases in both conditions. The balance was expressed using a scale of 0–10, with higher scores representing more stress on efficiency. Module C comprised a single-choice question that evaluated the conventional operating order around MHT. Implemental frequency of 10 randomly presented operation guidance were investigated in module D. The surgeons were requested to grade each guidance on a 4-point scale including “never,” “occasional,” “often,” and “always” according to the frequency with which they would apply in daily practice. Modules E and F were designed to evaluate the current knowledge of CVS in different levels. Module E focused on the basic concept of CVS. Then, apart from those who replied that they were unaware of CVS’s basic concept, the other participants were asked to select the exact CVS criteria from ten pieces of guidance relevant to the decrease of BDI in module F. The last module was optional, focusing on the number of BDI that participants had experienced in their career. The content filled in the questionnaire was not included in the final video scoring.

### Statistical analysis

Data were analyzed using IBM SPSS Statistics for Windows, version 23.0 (IBM Corp., Armonk, NY) and Microsoft Excel (Microsoft Corp., Redmond, WA). Data were presented as means and percentages. Differences between groups were determined by using the Mann–Whitney *U* test for numeric or ordinal variables, and using the *χ*^2^ test or Fisher’s exact test for categorical variables. The Cohn Bach's alpha of each module was evaluated. Multivariate logistic regression analysis relevant to the status of CVS was performed. In this analysis, variables were screened and selected according to the integration of cognition and execution parameters, and forwarding enrollment was based on the likelihood ratio test results. A *P* value of <0.05 was considered statistically significant.

ResultsA total of 169 LC surgical videos on procedures performed by 124 surgeons were collected, including 102 videos in the non-inflammatory group and 67 videos in the inflammatory group. Notably, 105 participants gave valid answers to the questionnaire. The participants were recruited from 67 different hospitals in the southwest regions of China.

### Baseline characteristics of participants

As shown in Table 1, over 70% of the surgeons enrolled in both groups of the present study were in their 30s, all of whom were males. Attending surgeons accounted for more than 50% of all participants. The post of an attending surgeon is like an intermediate professional title of doctors in China. Attending surgeons are board-certified and capable of operating LC on their own. The results showed that 56.9% of the surgeons in the non-inflammatory group had a caseload of above 200 LC, whereas 65.7% of the surgeons in the inflammatory group had a caseload of above 200 LC. However, there is no significant difference in all baseline characteristics between two groups.

**Table 1:** Baseline data of the participants.

	Non-inflammatory group (*N* = 102)	Inflammatory group (*N* = 67)	*P*
Age (years)			0.939
≤30	16 (15.7%)	10 (14.9%)	
31–40	74 (72.5%)	50 (74.6%)	
41–50	11 (10.8%)	7 (10.4%)	
≥50	1 (0.9%)	0 (0%)	
Gender			N/A
male	102 (100%)	67 (100%)	
female	0 (0%)	0 (0%)	
Professional qualifications			0.504
Resident	16 (15.7%)	7 (10.4%)	
Attending	52 (51.0%)	36 (53.7%)	
Vice-chief	31 (30.4%)	22 (32.8%)	
Chief	3 (2.9%)	2 (3.0%)	
LC caseload			0.159
≤50	19 (18.6%)	7 (10.4%)	
51–100	21 (17.6%)	10 (14.9%)	
101–200	7 (6.9%)	6 (9.0%)	
≥200	58 (56.9%)	44 (65.7%)	

LC, laparoscopic cholecystectomy; N/A, not applicable.

### Surgical video results

The results on surgical videos for all participants are given in Table 2. The inflammatory group had a significantly longer operation time (from establishing access to extracting the gallbladder and clearing the operative region) (46.98 min vs. 20.91 min, *P* < 0.001) and a longer surgical phase operation time (except EA) compared with those of the non-inflammatory group. MHT was the longest surgical phase in both groups. Out of the 169 surgical videos, 9 videos performed bail-out process directly; therefore, the other 160 surgical videos were used for subsequent CVS analysis. The overall CVS achievement rates were 18.18% and 9.84% for the non-inflammatory group and the inflammatory group, respectively. Although over 50% of the surgeons in both groups fulfilled the C3 criterion, the achievement rates of C1 and C2 were still low ranging from 11.5% to 27.9%. However, there was no significant difference in CVS achievement between the two groups.

**Table 2:** Analysis of laparoscopic cholecystectomy videos.

	Unit	Non-Inflammation group (*N* = 102)	Inflammation group (*N* = 67)	*P*
Total length	min	20.91 (20.08–26.48)	46.98 (30.53–58.85)	<0.001
Establish access		0.99 (0.65–1.28)	0.91 (0.60–1.40)	0.623
Adhesion lysis		0 (0–1.44)	3.50 (1.3–6.83)	<0.001
Mobilize the hepatocystic triangle		7.63 (5.41–10.07)	12.28 (7.47–17.03)	<0.001
Dissect the gallbladder from the liver bed		2.63 (1.70–4.03)	6.07 (3.75–16)	<0.001
Extract the gallbladder		1.38 (0.71–2.77)	2.30 (1.17–4.22)	0.008
Clear the operative region		2.00 (1.11–2.96)	5.07 (3.12–9.40)	<0.001
Idle time		2.69 (2.06–4.40)	8.15 (3.40–13.30)	<0.001
Duration outside Abdomen		1.00 (0.31–1.85)	1.70 (0.82–3.72)	<0.001
Frequency of camera pulling out	time	2 (1–4)	3 (1–5)	0.011
Times of surgical steps changing		18 (14–24)	29 (18–48)	<0.001
C1	freq			0.109
0		0(0%)	0(0%)	
1		82(82.8%)	44(72.1%)	
2		17(17.2%)	17(27.9%)	
C2				0.200
0		80(80.8%)	54(88.5%)	
2		19(19.2%)	7(11.5%)	
C3				0.906
0		7(7.1%)	8(13.1%)	
1		41(41.4%)	19(31.1%)	
2		51(51.5%)	34(55.7%)	
CVS Overall scoring	point	3 (2–3)	3 (2–4)	0.907
CVS achievement rate^a^		18/99 (18.18%)	6/61 (9.84%)	0.089
Undergo the bail-out process^a^		6/102 (5.9%)	9/67 (13.4%)	0.092
Undergo the bail-out process directly^a^		3/102 (2.94%)	6/67 (8.96%)	0.176^b^
Transfer to the bail-out process^a^		3/99 (3.0%)	3/61 (4.91%)	0.543^c^

^a^ Those who undergo the bailout process directly are not CVS scorable.

^b^ Continuity correlation was used.

^c^ Fisher’s exact test was used.

CVS, critical view of safety; C1, C2, and C3 represents the 1st, 2nd, and 3rd criteria of Critical View of Safety, or CVS.

### Questionnaire results

A total of 105 surgeons gave a valid response to the questionnaire. Among them, 39 participants each performed two LC surgical videos, both with and without severe inflammation. The dataset comprised 85 surgeons in the non-inflammatory group and 59 surgeons in the inflammatory group. The Cronbach's alpha of each part of the questionnaire ranged between 0.777 and 0.861 (see Supplementary Table 2).

**Figure 1:**
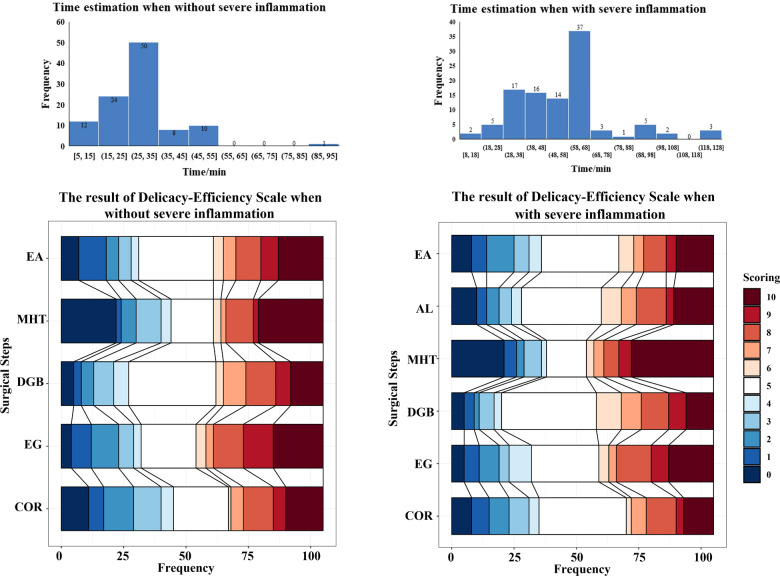
Time estimation and delicacy-efficiency scale. EA, Establish access; AL, adhesion lysis; MHT, mobilize hepatocystic triangle; DGB, dissect gallbladder from liver bed; EG, extract the gallbladder; COR, clear the operative region; the scoring of the Delicacy-Efficiency Scale ranged from 0 to 10, with a higher score (red blocks) representing more stress on efficiency and a lower score (blue blocks) representing delicacy. Five points (white block) represent keeping an equal balance between delicacy and efficiency in a particular step.

As shown in Figure 1, most surgeons reported that the estimated duration of LC with severe inflammation should be longer compared with the duration of LC without severe inflammation (58–68 min vs. 25–35 min). The results of delicacy-efficiency scale for different steps indicated that more surgeons preferred efficiency compared with delicacy in secondary surgical steps such as AL, DGB, and EG. Notably, a high number of surgeons chose either a fully efficient or a fully delicate approach when mobilizing the hepatocystic triangle, indicating a more controversial concept among surgeons performing such a critical task.

**Figure 2:**
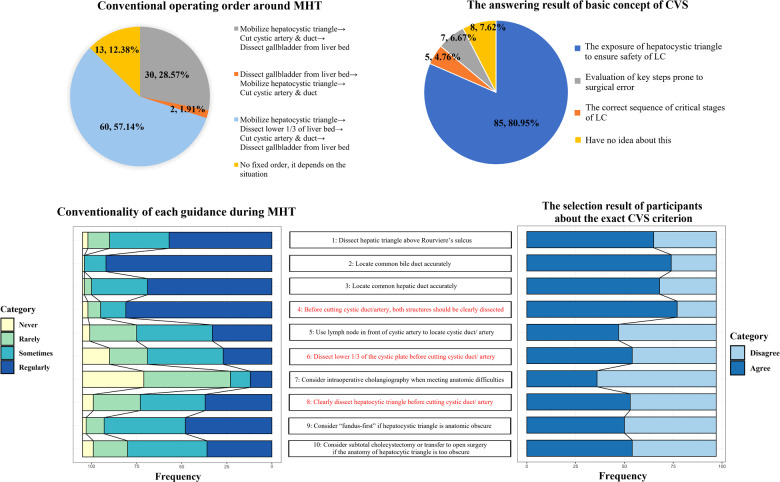
The operation habit and the cognition of CVS of participants. CVS, critical view of safety; MHT, mobilizing hepatocystic triangle; LC, laparoscopic cholecystectomy.

Although 80.95% of the surgeons had chosen the right concept of CVS as shown in Figure 2, only very few surgeons (3 in the non-inflammatory group and 3 in the inflammatory group, see Supplementary Table 2) could command all the three criteria in an error-free manner. The results in Figure 2 also showed that 57.14% of the surgeons claimed that they applied the routine surgical workflow that meets the requirement of CVS, whereas 42.86% of the surgeons applied other surgical workflows in daily practice. Further, the surgeons were presented with questions investigating the conventionality of each safety-proof guidance. The results also showed that 77.14% of the surgeons reported that they considered C3 routinely during surgery. However, only 25.71% and 35.24% of the surgeons reported that C2 and C1 were considered routinely during surgery, respectively. And the proportion of participants who routinely considered C2 and C1 during surgery shared similar trend with the proportion of participants harboring corresponding criterion as one of the requirements of achieving CVS (see Figure 2 and Supplementary Table 2).

**Figure 3:**
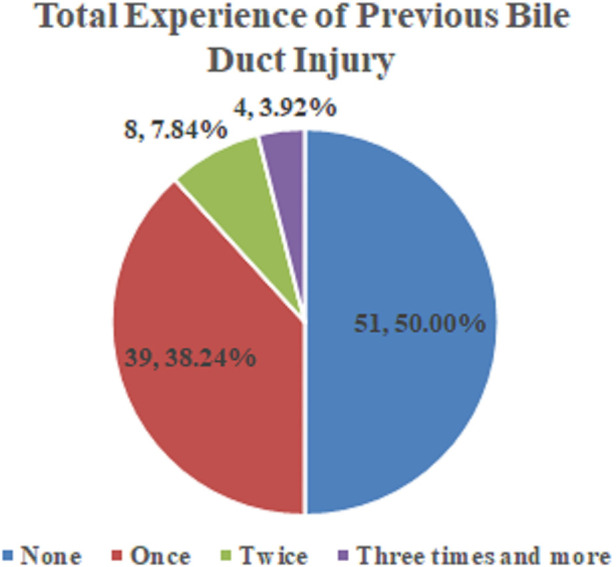
Total experience of a bile duct injury.

As shown in Figure 3, half of the surgeons (*N*= 51) who reported the experience of BDI (*N* = 102) admitted that they had experienced BDI. Details of these data are presented in Supplementary Table 2.

### Factors associated with low achievement of CVS

Multivariate logistic analysis through forwarding variant enrollment based on likelihood ratio test results was conducted to explore factors that affect the achievement of CVS. The results showed that unconventional surgical workflow in daily practice (OR: 12.372, *P* < 0.001) and a misunderstanding of the 2nd (OR: 8.917, *P* < 0.05) and 3rd (OR: 8.206, *P* < 0.05) criterion of CVS were associated with a low achievement of CVS (Table 3). Notably, the surgeons who did not choose the “fundus-first technique for hepatocystic triangle in obscure” as one of the CVS criteria had a better achievement of CVS (OR: 0.123, *P* < 0.01).

**Table 3:** Multivariate logistic regression differentiating odd ratio relevant to low achievement of critical view of safety (CVS).

Variables/factors	CVS scoring ≤2 points (low achievement)			CVS scoring 3 or 4 points (medium achievement)		
	B	Wald *χ*^2^ statistic	OR (95% CI)	B	Wald χ^2^ statistic	OR (95% CI)
Unconventional surgical workflow in daily practice	2.515	12.214	12.372 (3.018–50.711)^***^	1.084	2.471	2.957 (0.765–11.428)
Misunderstanding of the 3rd criterion of CVS	2.105	4.982	8.206 (1.293–52.097)^*^	0.981	1.142	2.667 (0.441–16.124)
Misunderstanding of the 2nd criterion of CVS	2.188	6.395	8.917 (1.636–48.601)^*^	2.283	7.817	9.811 (1.979–48.629)^**^
Not mistaken “fundus-first technique for hepatocystic triangle in obscure” as one of the CVS criteria	−2.093	9.437	0.123 (0.032–0.469)^**^	−1.577	6.335	0.207 (0.061–0.705)^*^

* *P* < 0.05.

** *P* < 0.01.

*** *P* < 0.001.

Reference outcome: CVS achieved.

Reference variables/factors: Conventional surgical workflow: “MHT→Dissect lower 1/3 of the liver bed→Cut cystic duct and artery → Dissect gallbladder from liver bed”, correctly regarding C3 as one of the CVS criteria, correctly regarding C2 as one of the CVS criteria, Not taking the “fundus-first technique for hepatocystic triangle in obscure” as one of the CVS criteria. C2 and C3 refer to the 2nd and 3rd criteria of CVS.

CI, coefficient interval; CVS: critical view of safety; OR: odds ratio.

## Discussion

Minimally invasive instruments and techniques have developed quickly; however, the incidence of BDI has not reduced in the present decade. Approximately 49% of surgeons experienced a major BDI in British Columbia, Canada (32). The results of our study indicated that 50% of surgeons had experienced BDI in their career. CVS is an intraoperative exposure technique initially introduced by Strasberg et al. (30) more than two decades ago. Several studies report that CVS is an accessible and effective technique to reduce the incidence of BDI (33–36). However, the achievement rate of CVS was significantly lower than expected and varied significantly in different institutions (25, 26, 37). Few studies have conducted a comprehensive analysis on the causes of low CVS achievement. Therefore, our study sought to explore factors that affect the achievement of CVS from both cognitive and executive aspects, showing that cognitive factors had a more significant effect on CVS compared with executive factors.

Currently, the achievement rate of CVS is not satisfactory. A total of 1,051 LC surgical videos from 31 surgeons were analyzed, and the findings indicated that only 9% cases fulfilled all three criteria of CVS (26). Moreover, no difference in CVS achievement was observed between low-severity cases and high-severity cases (26). Similarly, findings from a surgical improvement study conducted in France indicated an initial CVS achievement rate of 15.9% in 172 cases (25). In addition, the results from an analysis of a prospectively collected data deposited in the LC10000 database seen in Supplementary Figure 2 (https://lc10000.withai.com/) comprising 415 surgical videos showed that the overall achievement rate of CVS in the southwest regions of China was only 5%. In our study, although surgeons submitted surgical videos that they considered good, the actual achievement rates of CVS in the non-inflammatory group and the inflammatory group were 18.18% and 9.84%, respectively. These findings indicated that low achievement of CVS was a global problem, and therefore, further studies should explore ways to improve the achievement rate.

Some institutions have conducted research on surgeons' understanding of CVS, and the findings showed that the correct cognition rate of CVS was very low. A study conducted by Gupta et al. (38) indicated a significant discrepancy between self-cognition and actual cognition of CVS. In this study, most surgeons (88.3%) reported that they knew about CVS but only 11.5% knew about it correctly (38). A survey conducted in South America comprising 446 surgeons showed that the percentage of surgeons who correctly identified all three criteria of CVS was 21.8% (39). A large-sample survey conducted in the Netherlands, where CVS is routinely required during LC, indicated that although 98.2% of the surgeons claimed that they incorporated the CVS technique into daily practice, only 16.9% of the surgeons correctly selected all three criteria of CVS (40). In our study, although 80.95% of the surgeons knew about the basic concept of CVS, only a small percentage of these surgeons (3 in the non-inflammatory group and 3 in the inflammatory group, but one person submitted a video for each of the two groups) could command all three criteria in an error-free way. These findings indicated that the cognition rate of CVS was significantly lower than expected. In addition, the results indicated a significant difference in cognition at various regions/institutions.

In our study, questionnaires and analysis of surgical videos were first combined for in-depth research to explore the association between cognition and execution of CVS during LC. The results from the questionnaires indicated that 79.38% of the surgeons considered C3 as a criterion of CVS. However, only 54.64% of surgeons considered C1 and 55.67% of surgeons considered C2 as criteria of CVS. The results from an analysis of LC videos from these surgeons indicated that the achievement rate of C3 was significantly higher than that of C1 and C2. The results showed that surgeons had significant cognitive differences for each criterion of CVS, and these differences were ultimately reflected in their daily practice. Multivariate regression analysis was conducted in this study based on all parameters from the questionnaire and surgical videos. The results indicated that an incorrect understanding of essential CVS criteria was associated with a low achievement of CVS (scoring ≤2 points). Therefore, improving the cognition of CVS among surgeons may effectively increase the achievement of CVS.

Previous studies reported that education could improve the achievement of CVS. Findings from a previous study comprising 10 surgeons showed that although CVS was adequately achieved (score>4) by only two of the surgeons enrolled in the study initially, training five surgeons significantly increased the quality of CVS (from 1.75 points to 3.75 points, *P*< 0.05) (41). Nakazato et al. (27) reported that a structured curriculum for safe LC significantly increased the quality and frequency of achieving CVS. The study recommended routine application of this curriculum worldwide to improve the achievement rate of CVS (27). The increase of CVS achievement after education can decrease over time, indicating some decay in knowledge retention over time (42). Wong et al. (42) reported that continued educational interventions should be conducted to enhance long-term retention of knowledge. Moreover, current research on education does not provide regular feedback on the cognition of CVS in surgeons. Therefore, surgeons with a low understanding of CVS after group education should receive more individualized cognitive interventions.

Although education significantly improves the achievement rate of CVS, there are still 18%–58% LC procedures not achieving CVS (27, 43, 44). The effect of education on further improvement of CVS in some countries with high CVS penetration, such as the Netherlands, is very limited (44). Studies are currently exploring other approaches to improve the CVS achievement rate (19, 25, 43, 45–47). Mascagni et al. (25) demonstrated that performing a short intraoperative time-out significantly improved the achievement rate of CVS. Chen CB and colleagues (43) reported that a combination of focused education and intraoperative time-out could improve CVS scores and its knowledge. According to SAGES and other guidelines, indocyanine green is recommended to help avoid bile duct injury in complex situations where dissection is impossible to get a clear CVS view (19, 47). In addition, indocyanine green can also help our AI to identify the bile duct structure and its variation. At present, we are also carrying out some related studies and may combine anatomical recognition with indocyanine green for model optimization in the future. A few studies have reported promising application of AI techniques such as computer vision and deep learning for automated identification of CVS with high accuracy (26, 48–50), which can be utilized as a quality control system in future. These findings indicated that interventions and feedback in surgery were important for improving CVS achievement.

Although our study is the first comprehensive research deeply investigating the status of CVS in China from both cognitive and executive aspects, it has some limitations. First, because it is a voluntary surgical video contest, there is a limit to the sample size. The study has a small sample size and lacks global data, which may not be representative of the overall CVS achievement. Then, there is the Hawthorne effect in the analysis of videos. Surgeons selected surgeries they thought better during the video recruitment period to participate in the competition, so the CVS achievement rate may be higher than usual, which may not fully reflect the actual situation in daily practice. In addition, due to the gap between the number of doctors of different professional titles, the research results can only roughly reflect the overall situation but cannot accurately reflect the CVS completion of doctors of different professional titles. In the near future, we plan to promote SurgSmart to analyze and evaluate the daily practice of surgeons of varied professional titles in a wider range, so we can obtain more comprehensive and objective data.

In summary, low cognition and achievement rate of CVS is a global problem that must be solved. Additionally, cognitive factors have a more significant effect on CVS compared with executive factors. Thus, improving awareness of CVS and accurately grasping the requirement of the three criteria may effectively improve the achievement rate of CVS.

## Data Availability

The original contributions presented in the study are included in the article/**Supplementary Material**, further inquiries can be directed to the corresponding author/s.
