# Supplementary material for: Critical view of safety in laparoscopic cholecystectomy: A prospective investigation from both cognitive and executive aspects
Source: Front Surg. 2022 Aug 1;9:946917. doi: 10.3389/fsurg.2022.946917 (PMC9377448; doi:10.3389/fsurg.2022.946917)
Supplement: Supplementary file 1 [file Table_1_v1.docx]

Supplementary Material

## Supplementary Tables

**
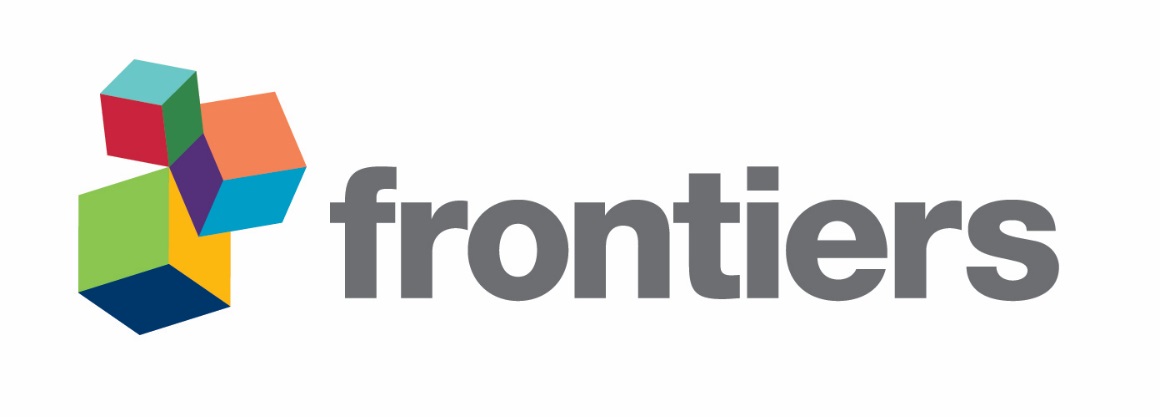
**

| Criteria | Scoring | | |
| --- | --- | --- | --- |
|  | 0 | 1 | 2 |
| 1. Remove excess tissue from the hepatobiliary triangle before disconnecting the duct | Not Achieved | Doubtful | Achieved |
| 1. Remove the lower third of the gallbladder bed before disconnecting the duct | Not Achieved | N/A | Achieved |
| 1. Before disconnecting the duct, the cystic artery and the cystic duct were both separated | Not Achieved | Doubtful | Achieved |

**Supplementary Table 1 Labeling Criteria of CVS**

**CVS Achieved when sum score not less than 5 points. N/A: Not Applicable, CVS: critical view of safety**

**Supplementary Table 2 Brief reports and Cronbach’s alpha of the overall questionnaire**

| Category | Investigation content | Unit | Non-Inflammatory Group | Inflammatory Group | Overall  (N=105) | Cronbach’s alpha | |
| --- | --- | --- | --- | --- | --- | --- | --- |
| Module A:  Time Estimation | LC without severe inflammatory | min | 30 (21—30) | 30 (24—35) | 30 (24—30) | | N/A |
|  | LC with severe inflammatory |  | 50 (40—60) | 60 (44—61) | 57 (40—60) | |  |
| Module B: Delicacy-Efficiency Scales* | EA when with no severe inflammatory | points | 5.38±3.07 | 5.39±3.34 | 5.52±3.20 | | 0.861 |
|  | MHT when with no severe inflammatory |  | 5.39±3.65 | 4.95±3.69 | 5.17±3.76 | |  |
|  | DGB when with no severe inflammatory |  | 5.68±2.69 | 5.27±2.78 | 5.69±2.73 | |  |
|  | EG when with no severe inflammatory |  | 5.62±3.12 | 5.90±3.45 | 5.90±3.23 | |  |
|  | COR when with no severe inflammatory |  | 4.95±3.25 | 5.07±3.24 | 5.00±3.61 | |  |
|  | EA when with severe inflammatory |  | 5.08±2.97 | 4.90±3.08 | 5.17±3.04 | |  |
|  | AL when with severe inflammatory |  | 5.68±2.97 | 5.07±2.91 | 5.50±3.01 | |  |
|  | MHT when with severe inflammatory |  | 6.18±3.77 | 4.90±3.88 | 5.61±3.92 | |  |
|  | DGB when with severe inflammatory |  | 5.68±2.48 | 5.36±2.67 | 5.76±2.60 | |  |
|  | EG when with severe inflammatory |  | 5.39±3.01 | 5.68±3.18 | 5.76±3.04 | |  |
|  | COR when with severe inflammatory |  | 5.33±2.79 | 4.56±2.79 | 5.10±2.94 | |  |
| Module C: Conventional operating order  around MHT | MHT→ Lower 1/3 Cystic Plate→ Cut cystic duct/ artery→ DGB/Others | Freq | 53/32 | 29/30 | 60/45 | | N/A |
| Module D: Conventionality of each guidance (Always/  Often/  Occasional/  Never) | 1. Dissect hepatic triangle above Rourviere’s sulcus |  | 44/27/11/3 | 32/19/6/2 | 57/33/12/3 | | 0.807 |
|  | 2. Locate common bile duct accurately |  | 73/11/0/1 | 49/9/0/1 | 92/12/0/1 | |  |
|  | 3. Locate common hepatic duct accurately |  | 54/27/3/1 | 38/18/2/1 | 69/31/4/1 | |  |
|  | 4. Before cutting cystic duct/artery, both structures should be clearly dissected |  | 65/12/6/2 | 41/11/5/2 | 81/14/7/3 | |  |
|  | 5. Use lymph node in front of cystic artery to locate cystic duct/ artery |  | 24/39/19/3 | 20/18/19/2 | 33/42/26/4 | |  |
|  | 6. Dissect lower 1/3 of the cystic plate before cutting cystic duct/ artery |  | 19/37/20/9 | 15/23/8/13 | 27/42/21/15 | |  |
|  | 7. Consider intraoperative cholangiography when meeting anatomic difficulties |  | 10/8/41/26 | 6/5/25/22 | 12/11/48/34 | |  |
|  | 8. Clearly dissect hepatocystic triangle before cutting cystic duct/ artery |  | 30/30/20/5 | 21/17/19/2 | 37/36/26/6 | |  |
|  | 9. Consider fundus-first if hepatocystic triangle is anatomic obscure |  | 37/42/6/0 | 30/20/7/2 | 48/45/10/2 | |  |
|  | 10. Consider subtotal-cholecystectomy or transfer to open surgery if the anatomy of hepatocystic triangle is too obscure |  | 27/40/14/4 | 24/19/12/4 | 36/44/19/6 | |  |
| Module E: Basic CVS understanding  F: CVS understanding^#^ (Regard  following standards as CVS criteria) | Knowing the basic concept/Wrong Answer/Not Clear |  | 67/11/7 | 51/5/3 | 85/12/8 | | N/A |
|  | Accurately commanding |  | 3/85 | 3/59 | 5/97 | | 0.777 |
|  | 1. Dissect hepatic triangle above Rourviere’s sulcus |  | 52/78 | 36/56 | 65/97 | |  |
|  | 2. Locate common bile duct accurately |  | 59/78 | 45/56 | 74/97 | |  |
|  | 3. Locate common hepatic duct accurately |  | 54/78 | 41/56 | 68/97 | |  |
|  | 4. Before cutting cystic duct/artery, both structures should be clearly dissected |  | 62/78 | 44/56 | 77/97 | |  |
|  | 5. Use lymph node in front of cystic artery to locate cystic duct/ artery |  | 41/78 | 22/56 | 47/97 | |  |
|  | 6. Dissect lower 1/3 of the cystic plate before cutting cystic duct/ artery |  | 44/78 | 29/56 | 54/97 | |  |
|  | 7. Consider intraoperative cholangiography when meeting anatomic difficulties |  | 35/78 | 15/56 | 36/97 | |  |
|  | 8. Clearly dissect hepatocystic triangle before cutting cystic duct/ artery |  | 45/78 | 27/56 | 53/97 | |  |
|  | 9. Consider fundus-first if hepatocystic triangle is anatomic obscure |  | 45/78 | 25/56 | 50/97 | |  |
|  | 10. Consider subtotal-cholecystectomy or transfer to open surgery if the anatomy of hepatocystic triangle is too obscure |  | 50/78 | 26/56 | 54/97 | |  |
| Module G: Experience of Intraoperative injury occurrence | CBDI |  |  |  |  | | N/A |
|  | None |  | 56 | 35 | 66 | |  |
|  | Once |  | 20 | 10 | 24 | |  |
|  | Twice and more |  | 2 | 4 | 4 | |  |
|  | Overall |  | 78 | 49 | 94 | |  |
|  | CHDI |  |  |  |  | |  |
|  | None |  | 64 | 42 | 77 | |  |
|  | Once |  | 12 | 5 | 13 | |  |
|  | Twice and more |  | 2 | 3 | 4 | |  |
|  | Overall |  | 78 | 50 | 94 | |  |
|  | Other injury |  |  |  |  | |  |
|  | None |  | 65 | 38 | 76 | |  |
|  | Once |  | 9 | 8 | 12 | |  |
|  | Twice and more |  | 1 | 2 | 2 | |  |
|  | Overall |  | 75 | 48 | 90 | |  |
|  | Overall |  |  |  |  | |  |
|  | None |  | 39 | 26 | 51 | |  |
|  | Once |  | 32 | 18 | 39 | |  |
|  | Twice |  | 6 | 4 | 8 | |  |
|  | Over Twice |  | 2 | 4 | 4 | |  |
|  | Overall |  | 79 | 52 | 102 | |  |

*: This scale ranges from 0 to 10 points, 0 represents fully delicacy during the respective step, 10 represents fully efficiency.

#：Those who claim that they do not clear about the basic concept of Critical View of Safety directly (non-inflammatory group = 7, inflammatory group = 3, overall = 8) were excluded from answering following questions relevant to contents of CVS criteria, so the number of candidates answering the next 10 questions are 78 and 56, respectively.

Abbreviations: EA, Establish access; AL, Adhesion Lysis; MHT, Mobilize hepatocystic triangle; DGB, Dissect gallbladder from liver bed; EG, Extract the gallbladder; COR, Clear the operative region; CVS: Critical View of Safety; LC, Laparoscopic Cholecystectomy; CBDI, common bile duct injury; CHDI, common hepatic duct injury;

**Supplementary Figure1. An example of the surgical report**

**
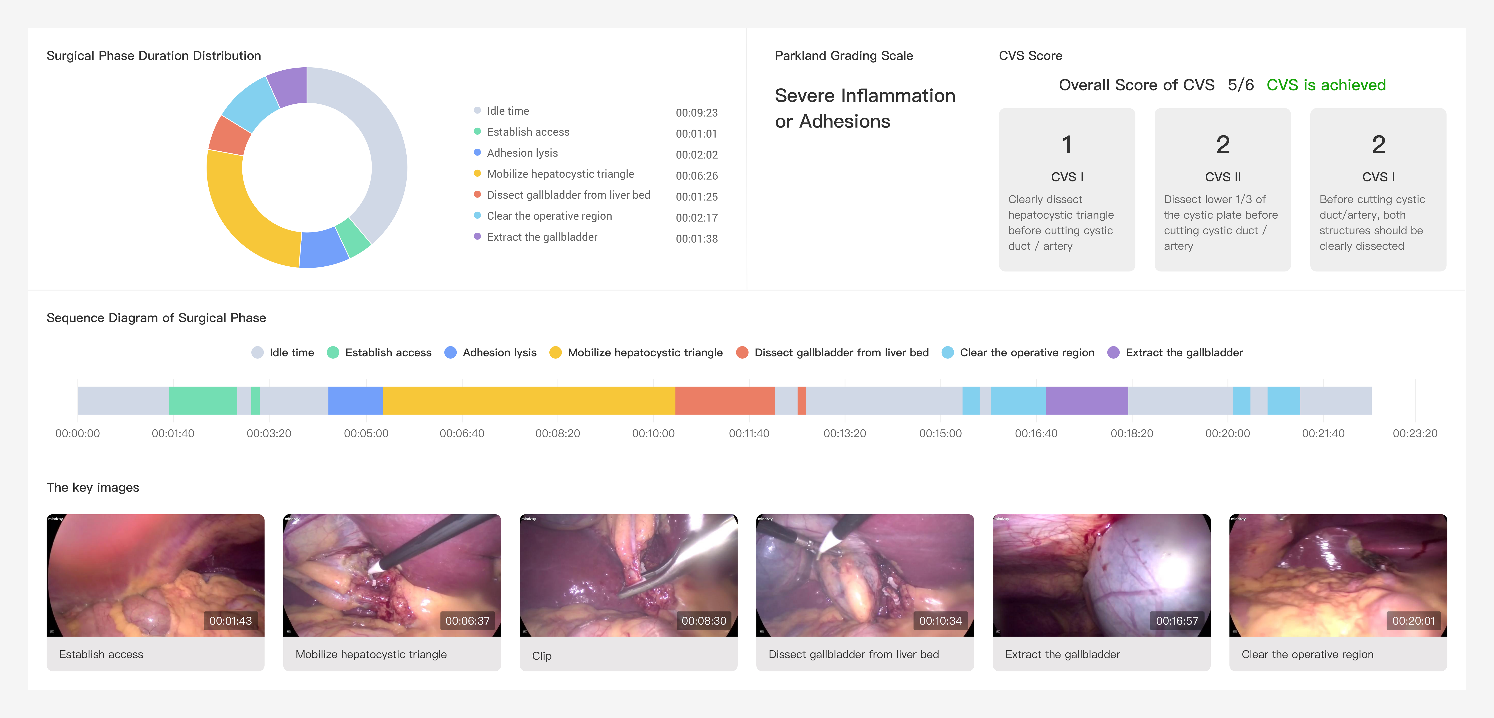
**

**Supplementary Figure2. An overview of LC10000 website**

**
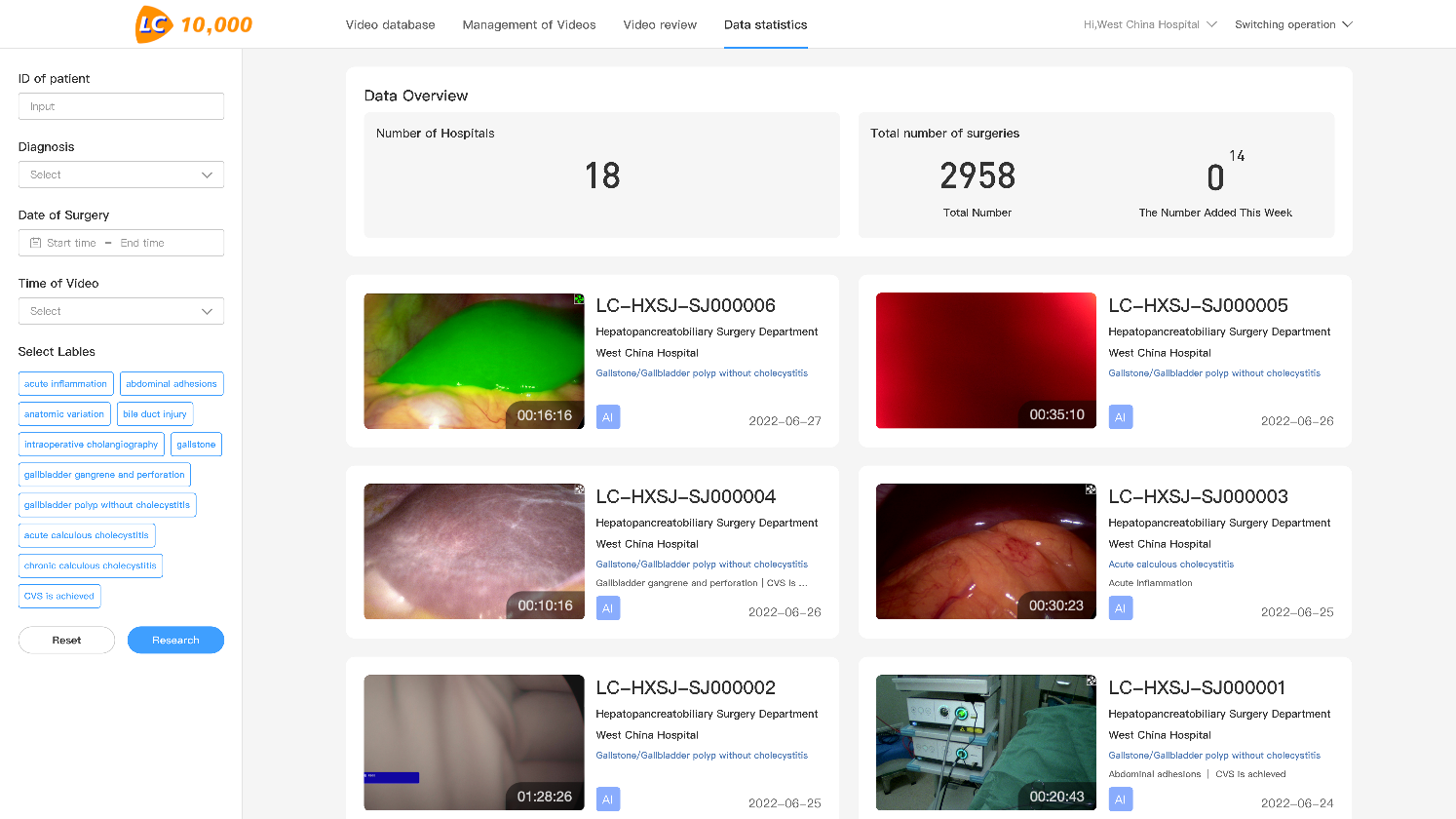
**

**Appendix——Overview of Questionnaire**

Introduction: Thank you for participating in this operation competition, we need your support and please take a few minutes to complete the questionnaire. This content will not be included in the score, so please take it easy.

Your name：_________  
The hospital where you work：____________

**A1.** What do you think is the appropriate time to get from establishing access to extracting the gallbladder and clearing the operative region when there is no severe inflammation? ______ (Please fill in the number from 0 to 120)

|  |  |
| --- | --- |
| time/  minutes | ________________________ |

**A2.** What do you think is the appropriate time to get from establishing access to extracting the gallbladder and clearing the operative region when there is severe inflammation? ______ (Please fill in the number from 0 to 180)

|  |  |
| --- | --- |
| time/  minutes | ________________________ |

**B1.** When there is no severe inflammation around the gallbladder, the most scientific proportion of delicacy and efficiency that you think：
             0   →   5   →  10  
delicacy: 100%→50% →0%
efficiency:  0% →50% →100%

for example：
   0   ：delicacy 100%，efficiency0%； 
   5   ：delicacy 50%， efficiency50%；
  10  ：delicacy 0%， efficiency100%

|  | 0 | 1 | 2 | 3 | 4 | 5 | 6 | 7 | 8 | 9 | 10 |
| --- | --- | --- | --- | --- | --- | --- | --- | --- | --- | --- | --- |
| Establish access (EA) | ○ | ○ | ○ | ○ | ○ | ○ | ○ | ○ | ○ | ○ | ○ |
| Mobilize hepatocystic triangle (MHT) | ○ | ○ | ○ | ○ | ○ | ○ | ○ | ○ | ○ | ○ | ○ |
| Dissect gallbladder from liver bed (DGB) | ○ | ○ | ○ | ○ | ○ | ○ | ○ | ○ | ○ | ○ | ○ |
| Extract the gallbladder (EG) | ○ | ○ | ○ | ○ | ○ | ○ | ○ | ○ | ○ | ○ | ○ |
| Clear the operative region (COR) | ○ | ○ | ○ | ○ | ○ | ○ | ○ | ○ | ○ | ○ | ○ |

**B2.** When there is severe inflammation around the gallbladder, the most scientific proportion of delicacy and efficiency that you think：

             0   →   5   →  10  
delicacy: 100%→50% →0%
efficiency: 0% →50% →100%

|  | 0 | 1 | 2 | 3 | 4 | 5 | 6 | 7 | 8 | 9 | 10 |
| --- | --- | --- | --- | --- | --- | --- | --- | --- | --- | --- | --- |
| Establish access (EA) | ○ | ○ | ○ | ○ | ○ | ○ | ○ | ○ | ○ | ○ | ○ |
| Adhesion lysis (AL) | ○ | ○ | ○ | ○ | ○ | ○ | ○ | ○ | ○ | ○ | ○ |
| Mobilize hepatocystic triangle (MHT) | ○ | ○ | ○ | ○ | ○ | ○ | ○ | ○ | ○ | ○ | ○ |
| Dissect gallbladder from liver bed (DGB) | ○ | ○ | ○ | ○ | ○ | ○ | ○ | ○ | ○ | ○ | ○ |
| Extract the gallbladder (EG) | ○ | ○ | ○ | ○ | ○ | ○ | ○ | ○ | ○ | ○ | ○ |
| Clear the operative region (COR) | ○ | ○ | ○ | ○ | ○ | ○ | ○ | ○ | ○ | ○ | ○ |

**C.** What is your conventional operating order around MHT?

| ○ Mobilize hepatocystic triangle → Cut cystic artery & duct → Dissect gallbladder from liver bed |  |
| --- | --- |
| ○ Dissect gallbladder from liver bed → Mobilize hepatocystic triangle → Cut cystic artery & duct |  |
| ○ Mobilize hepatocystic triangle → Dissect lower 1/3 of liver bed → Cut cystic artery & duct → Dissect gallbladder from liver bed |  |
| ○ No fixed order, it depends on the situation. |  |

**D.** During LC surgery, would you consider the following guidance?

|  | Never | Rarely | Sometimes | Regularly |
| --- | --- | --- | --- | --- |
| 1. Dissect hepatic triangle above Rourviere’s sulcus | ○ | ○ | ○ | ○ |
| 2. Locate common bile duct accurately | ○ | ○ | ○ | ○ |
| 3. Locate common hepatic duct accurately | ○ | ○ | ○ | ○ |
| 4. Before cutting cystic duct/artery, both structures should be clearly dissected | ○ | ○ | ○ | ○ |
| 5. Use lymph node in front of cystic artery to locate cystic duct/ artery | ○ | ○ | ○ | ○ |
| 6. Dissect lower 1/3 of the cystic plate before cutting cystic duct/ artery | ○ | ○ | ○ | ○ |
| 7. Consider intraoperative cholangiography when meeting anatomic difficulties | ○ | ○ | ○ | ○ |
| 8. Clearly dissect hepatocystic triangle before cutting cystic duct/ artery | ○ | ○ | ○ | ○ |
| 9. Consider fundus-first if hepatocystic triangle is anatomic obscure | ○ | ○ | ○ | ○ |
| 10. Consider subtotal-cholecystectomy or transfer to open surgery if the anatomy of hepatocystic triangle is too obscure | ○ | ○ | ○ | ○ |

**E.** What do you think is the correct meaning of CVS? (Single choice)

- The exposure of hepatocystic triangle to ensure safety of LC
- Evaluation of key steps prone to surgical error
- The correct sequence of critical stages of LC
- Have no idea about this

**F.** Which of the following guidance do you think CVS contains? (multiple choice)

- Dissect hepatic triangle above Rourviere’s sulcus
- Locate common bile duct accurately
- Locate common hepatic duct accurately t
- Before cutting cystic duct/artery, both structures should be clearly dissected
- Use lymph node in front of cystic artery to locate cystic duct / artery
- Dissect lower 1/3 of the cystic plate before cutting cystic duct / artery
- Consider intraoperative cholangiography when meeting anatomic difficulties
- Clearly dissect hepatocystic triangle before cutting cystic duct / artery
- Consider “fundus-first” if hepatocystic triangle is anatomic obscure
- Consider subtotal-cholecystectomy or transfer to open surgery if the anatomy of hepatocystic triangle is too obscure

**G.** How many of the following injuries have you experienced during LC? (Optional)

|  | None | Once | Twice | More than twice |
| --- | --- | --- | --- | --- |
| Bile duct injury | ○ | ○ | ○ | ○ |
| Hepatic duct injury | ○ | ○ | ○ | ○ |
| Vascular injury | ○ | ○ | ○ | ○ |
| Other injury | ○ | ○ | ○ | ○ |
